# Supplementary material for: Short tandem repeat variants are possibly associated with RNA secondary structure and gene expression
Source: PLoS One. 2025 Jun 18;20(6):e0326355. doi: 10.1371/journal.pone.0326355 (PMC12176206; doi:10.1371/journal.pone.0326355)
Supplement: S1 File — Each is characterized by gene feature, sequence motif, and amino acid motif. (PDF) [file pone.0326355.s001.pdf]

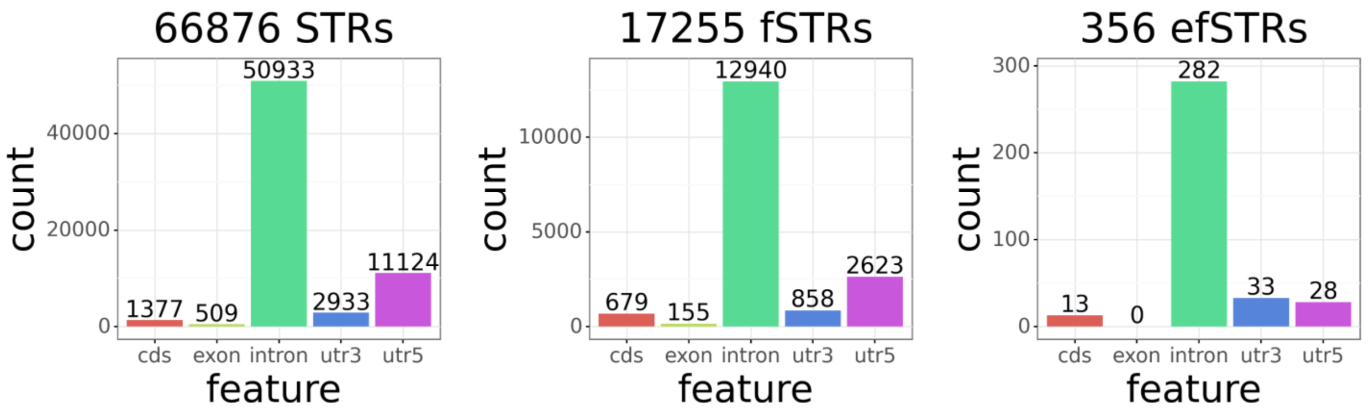

Figure S1 – Characterization of STRs, fSTRs, and efSTRs by gene feature.

Expanded characterization of STRs, fSTRs, and efSTRs

A genome wide collection of 66,876 transcribed short tandem repeats (STRs) were considered in this study; over 25% of these (26.1%) affect RNA folding (fSTRs). Effects of array length variants on RNA folding were inferred with ViennaRNA and bpRNA-align (see main text). Association with gene expression was established for 356 fSTRs (efSTRs). Characterization of STRs, fSTRs, and efSTRs by gene level annotation is shown in figure S1. Characterization by sequence motif and amino acid motif is shown in figure S2 and figure S3, respectively. Coding regions are overrepresented among fSTRs (figure 2a main text). The tally of amino acid motifs (figure S3) reveals a general hydrophilic tendency with the exception of proline (P), leucine (L), glycine (G), and alanine (A). The well know poly-glutamine motif is common among fSTRs and efSTRs. This result is bolstered by figure 4D (main text) which suggests CAG insertion variants cause extensive transcript level secondary structure alterations. In particular, CAG repeats conserve right-handed stems (R), left-handed stems (L), and ends (E) while departing from other structural motifs.

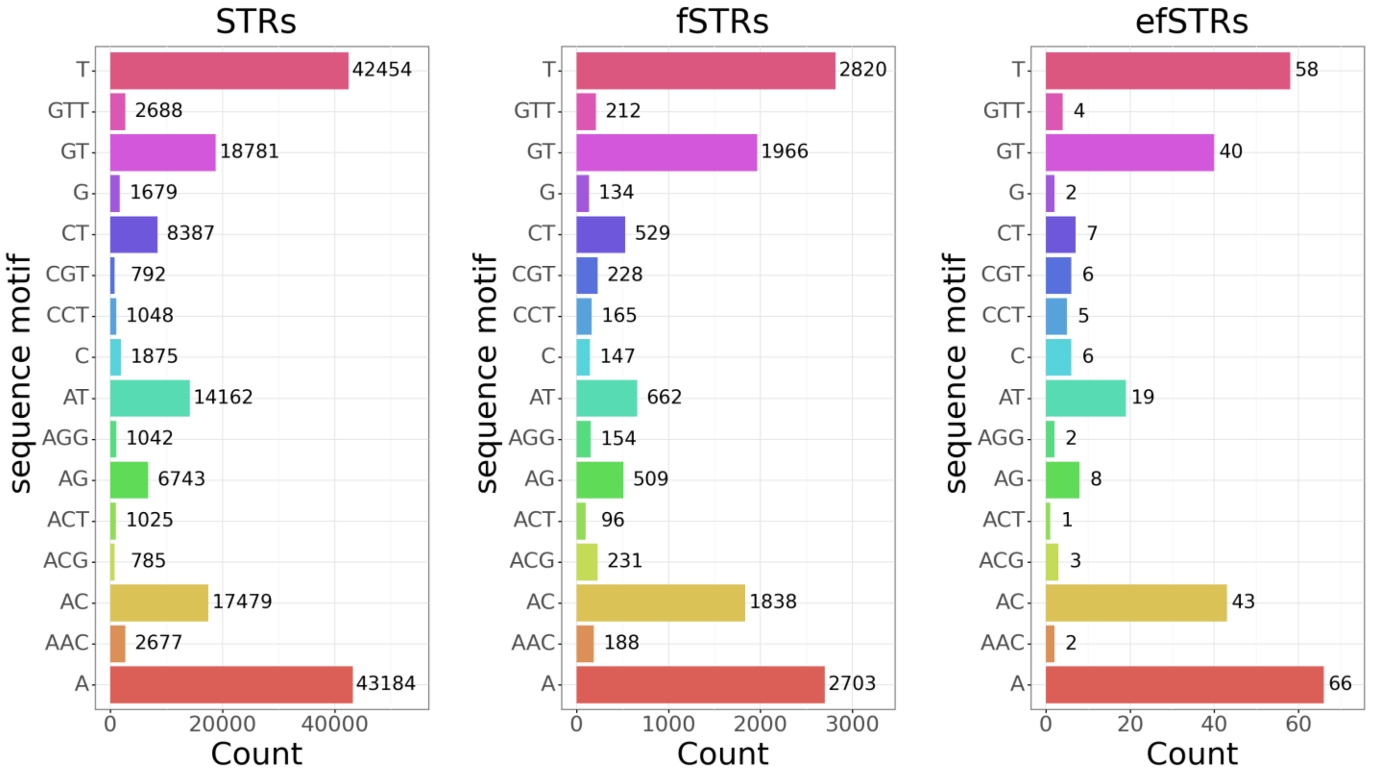

Figure S2 – Characterization of STRs, fSTRs, and efSTRs by sequence motif.

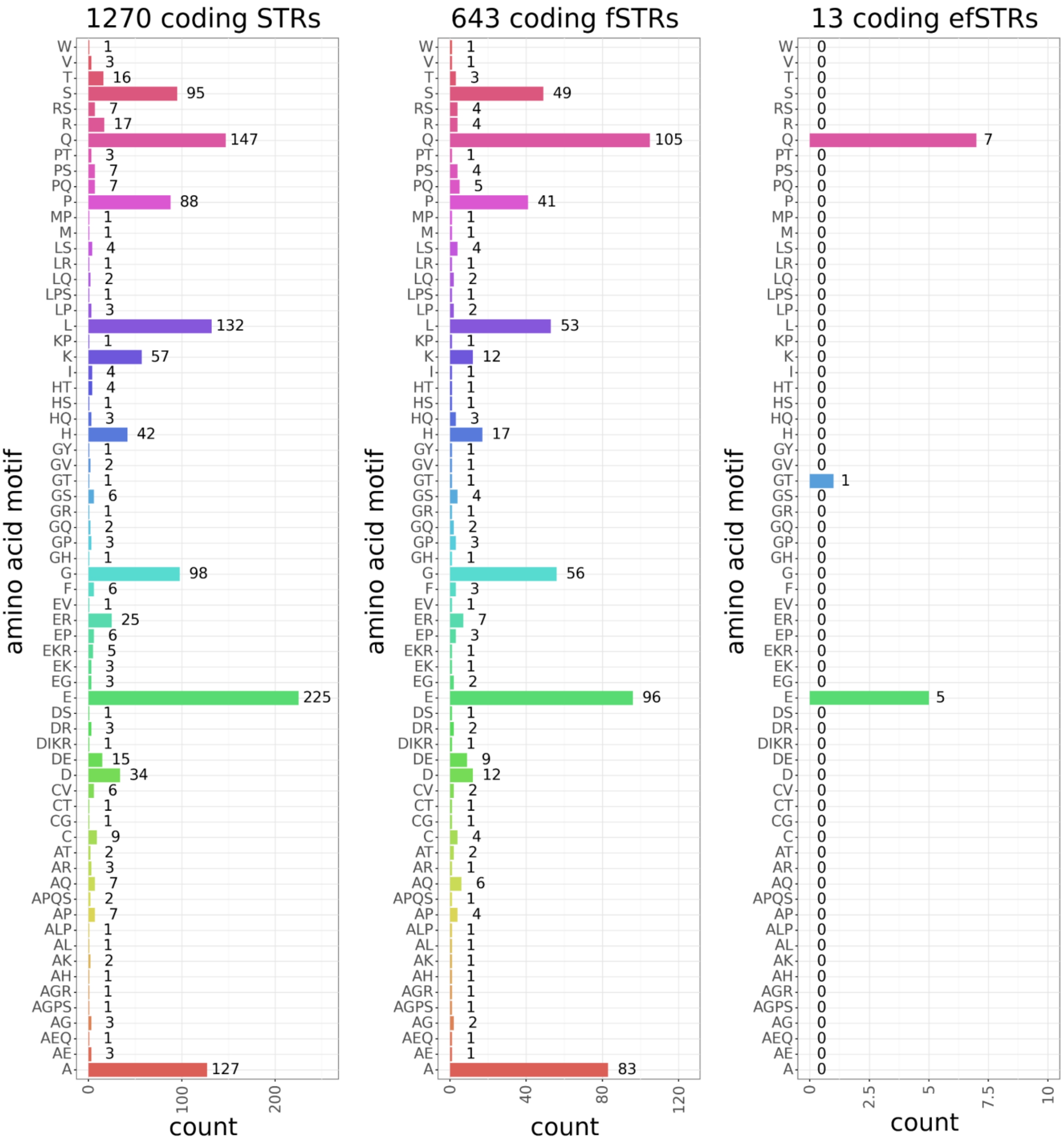

Figure S3 – Characterization of coding STRs, fSTRs, and efSTRs by amino acid motif. The tally of amino acid motifs reveals a general hydrophilic tendency with the exception of proline (P), leucine (L), glycine (G), and alanine (A). The well know poly-glutamine motif is common among fSTRs and efSTRs.
