# Supplementary material for: Short tandem repeat variants are possibly associated with RNA secondary structure and gene expression
Source: PLoS One. 2025 Jun 18;20(6):e0326355. doi: 10.1371/journal.pone.0326355 (PMC12176206; doi:10.1371/journal.pone.0326355)
Supplement: Attachment — Submitted filename: Response_to_reviewers.pdf [file pone.0326355.s003.pdf]

Nicholas Kinney, PhD
PO BOX 121
Sweet Briar, VA 24595
Phone: 301.338.1181
Email: nkinney@sbc.edu

April 28, 2025

Karthikeyan Thiyagarajan, PhD
Academic Editor, PLOS One
RE: POINT BY POINT RESPONSE TO PEER REVIEW COMMENTS (1ST REVISION)

Dear Dr Thiyagarajan:

In the pages that follow we respond point-by-point to each of the reviewer comments concerning our manuscript: "SHORT TANDEM REPEAT VARIANTS ARE ASSOCIATED WITH RNA SECONDARY STRUCTURE AND GENE EXPRESSION".

We appreciate the reviewer comments which have helped to improve our manuscript. Two reviewers suggested expanding our discussion of possible experimental validation. In response, we have added passages discussing the possibility of CLIP, Ribo-seq, SHAPE-seq, and DMS-seq. Two reviewers suggested clarifying statistical considerations. We have addressed these comments with a dedicated section in our methods. Several comments suggested simplifying figure 1: we have made an effort to do so by removing exemplars in 1A and labeling cluster assignments in 1B. Minor changes have been made throughout the manuscript to improve readability. The changes have improved the work and we thank all reviewers for their feedback. We would also like to clarify sources of funding as requested by the journal:

• The authors received support for publication fees from the Sweet Briar College Faculty Advancement Committee

• The funders had no role in study design, data collection and analysis, decision to publish, or preparation of the manuscript.

• The authors did not receive a salary from any funders.

We are excited to share our revised manuscript with the scientific community and look forward to hearing back. There are no related articles published or under consideration. If there are any specific questions that need a conversation, my cell is 301 338-1181.

Sincerely,

Nicholas Kinney

Dr. Nicholas Kinney
Assistant Professor of Biology, Sweet Briar College

Editorial comments:

I appreciate your work concerning the association of short tandem repeats in RNA folding and gene expression, specifically referring to the context of genetic disorders. However, there are already several studies available concerning the concept of tandem repeat-associated diseases; please check these specific articles: https://pmc.ncbi.nlm.nih.gov/articles/PMC3960014/ and https://pubmed.ncbi.nlm.nih.gov/16254211/. How is your study different from those that exist already?

We appreciate the overall positive feedback from the reviewers and editor of Plos ONE. We hope that our study has broad relevance in the context of genetic disorders as pointed out by the editor. The specific studies pointed out by the editor are valuable contributions that we were unaware of. The first provides a comprehensive review of mechanisms linking tandem repeats to various disease and phenotypic states. Two passages from that review suggest a link between RNA structure of transcribed repeats and gene expression. The following passage regarding the FMR1 is a good example:

> *Secondary structures, particularly when they occur in the 5′ UTR, are associated with problems with ribosome scanning. It is thus reasonable to think that the translation deficit is related to the ability of CGG-RNA to form stable RNA hairpins (Handa et al. 2003; Zumwalt et al. 2007).*

This specific example supports the plausibility of our hypothesis that many more transcribed STRs affect RNA structure and possibly gene expression. However, this example also clarifies the novelty of our study. In particular, ours is conducted at scale using a whole exome sequencing and gene expression data. These types of datasets did not exist when the aforementioned studies were conducted; consequently, they focus on a relatively small collection of high penetrance repeat expansions. In addition, the bpRNA-align clustering algorithm – used extensively in our approach – was published earlier this year.

We hope that our use of high-powered datasets and state of the art RNA clustering sheds light on the subtle effects that repeat expansion can have on gene expression.

It is also better to discuss from a thermodynamic point of view about the fold change due to variations of STRs.

I think I understand, to be sure I'd like to provide my interpretation of the feedback. Briefly, we predict minimum free energy (MFE) structures for transcribed STRs. Whenever the predicted structures differ, we use an RNA-seq dataset to check for differences in gene expression.

In reality, the transcribed repeats fold into an ensemble of structures approximately 1k_bT around the MFE structures. Consequently, some of the structures we predict may represent thermodynamically overlapping ensembles. However, we do not expect thermodynamically overlapping ensembles to support significant differences in gene expression. Thus, different structures that occupy similar thermodynamic ensemble naturally get weeded out from our results when we check for differences in gene expression. In the interest of accessibility – and reaching a wide audience – we do not delve into these thermodynamic considerations. One of the advantages of open peer review is that there will be a track record of this feedback and our decision making summarized in this correspondence.

Also, the intervening STRs in terminal inverted repeats and palindromes may be crucial to affect their hairpin loop kind of structure formation associated with miRNA and transposable elements.

Indeed, terminal inverted repeats and palindromes harbor some interesting biology. However, we are committed to focusing exclusively on short tandem repeats (STRs). This decision takes into consideration the biology of STRs ensuring that the regions we consider are more or less on equal footing; i.e. a control variable.

To expand on this point, recall that STRs are prone to unequal crossing over and strand slip replication: both precursors to array length mutations. Our analysis then characterizes broad differences based on repeat motif. Suppose we take into consideration palindromes and inverted repeats. Would we be looking for array length polymorphisms? What is the repeat motif for a palindrome or inverted repeat? It would be hard to compare these other types of repetitive elements on equal footing with STRs.

Our approach can certainly be expanded to other classes of repeats and their variants but considering all these possibilities is beyond the scope of this work. We have made minor changes to the discussion so that readers understand the approach is not limited to STRs. Indeed, we hope that the work offers new avenues of research for our colleagues.

Further, you could consider the role of methylation-related specific genes, discussing the methylation and demethylation processes during the suppression and expression of the genes.

It's a great idea to discuss methylation which may affect gene expression for CpG repeats. We now mention this in the discussion. We offer three additional thoughts on the matter. First, our study is focused on transcribed STRs. Since RNA transcripts are not methylated, our results for CpG repeats should be robust to this DNA specific modification. Second, fSTRs are associated with gene expression using an RNA-seq dataset. Any epigenetic silencing of CpG repeats should reduce RPKM values and eliminate any potential association with gene expression. In other words, it's unlikely that CpG methylation will lead to false positive fSTRs associated with gene expression. Third, we want to keep the work focused on understanding how transcribed STRs affect RNA secondary structure. Once we open the door to DNA modifications and different classes of repeats (see previous response) our results will become harder to interpret. Moreover, we do not wish to burden the reader with attempts to deconvolve the affects DNA modifications and repeat classification. These topics are better explored in separate works.

Besides, I have some additional comments and suggestions: Have you predicted Z-DNA and H-DNA caused by short tandem repeats with your study? Because I didn't see more detail in the result and discussion.

We thought about Z-DNA and H-DNA folding and considered making these predictions but chose not to for reasons stated in our previous two responses. Undoubtedly, some STR array length variants will affect the secondary structure of DNA: Z-DNA, H-DNA, cruciform DNA, and β-DNA, to name a few. However, our work is committed to understanding how STR array length variants affect the secondary structure of RNA, not DNA. In addition, our approach makes use a state-of-the-art RNA clustering algorithm which is not easily applied to DNA. Therefore, it's unclear how DNA secondary structure would fit into our proposed methodology. Nevertheless, we have discussed this idea at the end of the paper in hopes that readers will see this as low hanging fruit.

Hydrophilic amino acids are perhaps more tolerated due to the expansion of specific STR stretches, according to Katti et al., 2001, as you have cited, so please verify whether over-represented?

Although all the STRs we consider are transcribed, relatively few are in coding regions. The 356 fSTRs associated with gene expression include 13 in coding regions: five are poly-glutamic acid, seven are poly-glutamine, and one is a hexamer threonine-glycine repeat. Thus, the 13 translated fSTRs associated with gene expression all code for hydrophilic amino acids. If we look at the broad set of 17,255 fSTR (not necessarily associated with gene expression) we do find hydrophobic amino acids but hydrophilic amino acids still seem to be over-represented. While this is a bit anecdotal it seems to support Katti et al., 2001. If necessary, we can formulate and test an appropriate null; of course, our entire dataset will be released upon publication.

Figure 1B is not clear about the cluster assignments without providing any cluster combination grouping names, from a genetic point of view, the STR-induced variations of the cluster perhaps representing alternative alleles. Please explain better and make Figure 2B self-explanatory. You could represent AA, AB, BB like this or using any specific conventions.

Always a good idea to maximize accessibility. We have updated figure 1 for easier interpretation.

Have you compared the normal STR besides the fSTR with expanded stretches to the wild ones (normal STR) that affect gene expression? For instance, a similar concept in plants was explained before in plants; please see this article: https://www.nature.com/articles/ng822z

Figure 2 in the main text is focused on characterizing the 17,255 fSTRs. It seems like a good idea to compare fSTRs and non-fSTRs side-by-side. We have added supplementary material that offers expanded characterization of all transcribed STRs, fSTR, and non-fSTRs.

As you have mentioned, "fSTRs by unit are harder to interpret" because, in real time, the expression of the genes depends on many factors; however, the specific expansion of SSR due to polymerase slippage-derived errors, etc., may have a role in altered expression in exceptional cases with genetic diseases, etc. But in general, if you see even the SSR motifs from specific geographical isolates, perhaps they are more conserved than others. So, the study with fSTR with differential gene expression is perhaps more applicable to the specific individuals than others; in this case, you may need to interpret your results from Figure 3 carefully, as there are contradicting points about the accessibility.

If I understand correctly, you are suggesting the accessibility of transcribed STRs is itself a phenotype that may have more to do with individual genetic variation than the array length of the STR. This idea is certainly possible. It’s also possible that the accessibility of the MFE structures we predict for transcribed STRs differ somewhat from the structures that exist under true physiological conditions. To be more precise, figures 3 and 4 now clearly state that accessibility is for predicted MFE structures.

Even if you speculate about poly AT and poly TA pairing, the folding and unfolding need specific temperature-dependent variations with specific examples, like fSTR information comparing the housekeeping genes from humans to apes, etc.

Thermodynamic considerations were taken very seriously in our study design despite the lack of thorough documentation in our methods. This issue has now been addressed with a new passage at the end of the methods section. The reason for the omission is that the ViennaRNA the energy model incorporates experimentally derived nearest-neighbor parameters under physiological conditions (typically 37°C and standard ionic strength), providing a biologically relevant approximation of the RNA's likely folding state. Temperature-dependent variations are mitigated by the fact that we are making predictions at constant physiological conditions.

A related thermodynamic issue has to do with how well the MFE structural predictions represent the entire ensemble of structures that will exist for each fSTR variant. We addressed this in a previous comment and reiterate our thought process here. We only predict MFE structures for each transcribed STR variant; in reality, each folds into an ensemble of structures approximately 1k_bT around the MFE structures. Therefore, it's conceivable that different MFE structures are actually part of similar overlapping ensembles of structures that mitigate any biological effects. Use of MFE structures without taking into consideration the entire ensemble of structures could lead us to identify false positive fSTRs. This argument leads to the following question: why don't we predict ensembles for each transcribed STR variant?

We suggest that associations between fSTRs and gene expression offer a form of validation. Essentially, we agree with the editor that thermodynamic fluctuations add a layer of complexity. However, at the end of the day we are able to associate predicted structural differences to real changes in gene expression.

Comparisons to housekeeping genes in humans and apes may offer an interesting way to check if the fSTR effects on gene expression are biologically relevant. For example, are the RPKM changes associated with fSTRs greater than the variation we see for housekeeping genes within and between species? It's an interesting idea but I would be careful assuming that small RPKM differences for some genes are not meaningful. These genes are part of different pathways; for some, tiny changes may be biologically significant. We will consider this analysis in a future work

Figure 4: Is this figure derived from the similarity correlation score for the tallied pairs of fSTR alleles? You have shown the associations of different fSTR between shorter and longer variants. Is it with a CAG fSTR that more associations with gene expression occur? And how would you infer the effect due to the stability of the folding between the shorter and longer variants?

This interpretation is mostly correct. Generally, a folded RNA transcript has a variety of secondary structure elements such as internal loops, bulges, hairpins, and multiloops. Figure 4 summarizes how these secondary structure assignments change for fSTR insertion variants.

Not all CAG repeats are fSTRs, but they are over-represented; this is overshadowed in figure 2 due to the large number of monomer fSTRs; and, it is not our intent to focused on CAG repeats. On the other hand, we recognize that CAG repeats have an established role in disease and included them in figure 4 to reach a wider audience. The stability of fSTRs may make for interesting analysis but is beyond the scope of this work which is focused on how to identify them. Conceivably, the CAG motif forms relatively stable secondary structures due to its CG content.

You have discussed more about the accessibility, but why didn't you consider the stability of RNA structure due to the variations of motifs as you have compared through the bpRNA-align?

It's a good idea to consider the stability of the RNA structures. We omitted this analysis because it seems just outside the scope of our work. The hypothesis of our work is that some transcribed STR variants effect RNA structure with associated effects on gene expression. Once this hypothesis is supported, we offer an explanation based on RNA accessibility and the types of motifs that affect accessibility. The idea is that accessibility influences rates of translation, splicing, and RNA-protein binding affinity. The stability of secondary structures may contribute to gene expression; but, it's harder to make a direct link without significantly expanding the scope of our study.

You have also mentioned the use of the RNAfold program to predict thermodynamic ensembles. Why didn't you show the relevancy of the structural change with a thermodynamic point of view for specific sequences that are varying for specific fSTRs?

We are using the ViennaRNA package to predict MFE secondary structures which uses an experimentally derived energy model under physiological conditions. Therefore, the secondary structures we predict for transcribed STR variants are relevant from a thermodynamic point of view. When we identify fSTR variants in different clusters we check for associations with gene expression. As discussed in a previous response, it is conceivable that the thermodynamic energy barrier between clusters is low such that different MFE structures are essentially part of the same ensemble. In these cases, we do not expect to see an association with gene expression. Indeed, we identify 17,255 fSTRs but only 356 associated with gene expression. While interesting, consideration of thermodynamic ensembles of structure would probably not eliminate false positive gene expression associations. For this study it is better to keep the approach simple enough that others can make improvements where they see fit.

Large tandem repeats are also known to be associated with diseases. How do you compare them with STRs, and which major genes were focused on for differential gene expression through DESeq2?

Often large repeats exceed the 100bp read length making variant calling difficult. There may be some modern variant callers that can make educated guesses for large repeats but doing so would introduce heterogeneity into our dataset. It's also worth reiterating that STRs are prone to strand slip replication and unequal crossing over. Other classes of repeats may mutate for different reasons. This is another source of dataset heterogeneity we intentionally avoid by sticking to STRs.

I suggest you revise your manuscript as suggested by me and reviewers, make the contents more succinct, please try to avoid speculations, and please make the figures as self-explanatory. I also suggest comparing the species from other kingdoms; even you could compare specific housekeeping genes forms from prokaryotes to see whether there are any folding and gene expression differences due to orthologous gene comparisons, and you can also compare the specific duplicated or paralogous variations within a specific species or even with the specific genes from humans.

We look forward to sharing the revised manuscript. Unfortunately, branching out to other kingdoms would require a complete reference genome as well as paired genome sequencing and gene expression data for a large number of individuals. This analysis will probably have to wait for a future study.

Reviewer #1:

statistical analysis maybe has not been performed appropriately and rigorously, for example following: Figure 3 – Effects of fSTR variants on RNA accessibility. Accessibility is inferred from the tally of unpaired bases using ViennaRNA. Results of three types are obtained: (a) accessibility increases with allele length; (b) accessibility decreases with allele length; and (c) accessibility is not associated with allele length. (d) reverse complementary sequences tend decrease RNA accessibility.

This is a good point. We have updated the figure legend with correlation coefficients and p-values for each subplot. Our overall conclusion remains unchanged. Insertion variants for reverse complementary motifs tend to decrease transcript accessibility; on the contrary, insertion variants for non-reverse complementary motifs tend to increase transcript accessibility. The following plot of correlation coefficients for high-coverage experimental motifs offers additional support:

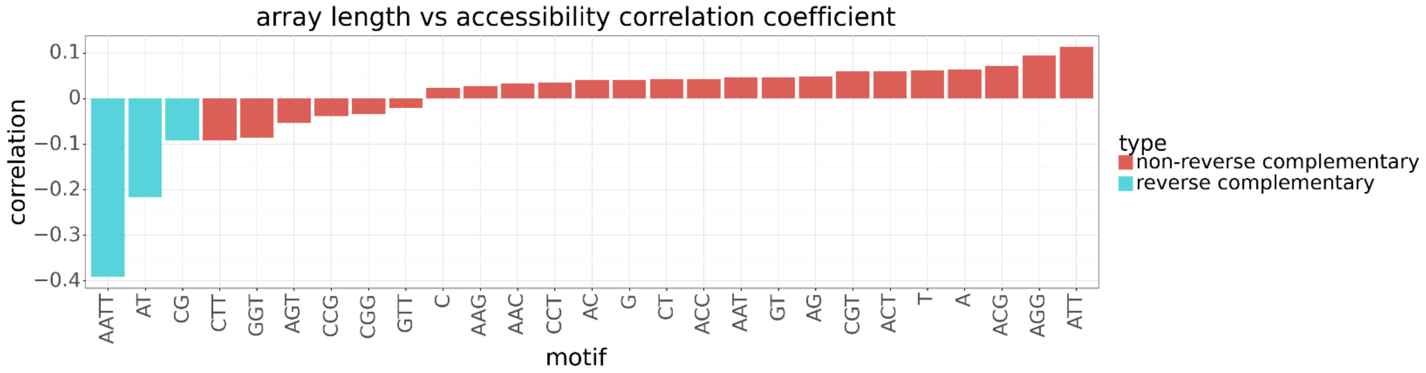

Reviewer #2:

Thank you for the opportunity to review the manuscript: "Short tandem repeat variants are associated with RNA secondary structure and gene expression". The manuscript presents a comprehensive analysis of the impact of short tandem repeat (STR) variants on RNA folding and gene expression. The study leverages data from the 1000 Genomes Project and ViennaRNA to identify transcribed STRs that affect RNA secondary structure, providing valuable insights into the role of STRs in gene regulation. The strengths of the manuscript include its robust computational approach, the large dataset used for analysis, and the novel findings regarding the association between STR variants and RNA accessibility.

We appreciate the positive feedback.

However, the manuscript could benefit from a more detailed discussion on the biological implications of the findings and potential experimental validation to support the computational predictions. Additionally, the characterization of STRs by motif length and gene level annotation, while thorough, could be expanded to include more diverse genomic contexts. Overall, the study makes a significant contribution to the understanding of STRs and their effects on RNA structure and gene expression, paving the way for future research in this area.

This is a reasonable suggestion and one which we also received from the editor. We now have supplementary material that offers expanded characterization of fSTR and non-fSTR.

What parameters were used to identify STR variants in 2,529 samples from the 1000 genomes project? I cannot find this information in the MS. Please specify the methods and tools used for STR variant identification. Additionally, describe in detail the lengths and variations of the tandem repeats considered as STRs in this study.

We used the same dataset in a previous publication and refer the reader to that publication for details. In retrospect, it is a good idea to simply include that information here. Of course, our dataset and analysis scripts will be released on publication.

There was any association with STRs of different repeat length?

This is an interesting idea. I more or less assumed that fSTR clusters are binning the repeat length. This is equivalent to saying array length differences within clusters are smaller than array length differences between clusters. This leads to speculation about how various array length thresholds may coincide with rates of translation, splicing, and RNA-protein binding to transcribed STRs. It is easy enough to check array length differences within and between cluster. Allele differences between cluster exceed those within clusters for 14,888 out of 17,255 fSTRs. The same can be said for 270 out of 356 fSTRs associated with gene expression.

Please provide all raw data including the coordinates of tested genes and STRs.

We look forward to it!

Would it be possible for G-quadruplexes to form in G-rich STRs?

I suppose it's possible; this was an idea reiterated by the editor. For clarity we decided to limit this work to analysis of RNA-secondary structures. It is possible to extend our approach to DNA secondary structures: G-quadruplexes, Z-DNA, H-DNA, cruciform DNA, and β-DNA. This would entail predicting DNA secondary structures for STR variants; clustering based on presence or absence of the predicted structures; and associating any differences with gene expression. We now make this point clear in the discussion and invite readers to use our dataset as a starting point.

Is the representation of STRs different in gene regions compared to non-gene regions? Please provide statistics on the occurrence of individual STRs.

For this work we focus exclusively on transcribed STRs; therefore, all are in gene regions. Also, the collection of variants we use comes from whole exome sequencing of 1000 Genomes Project Samples reinforcing our focus on gene regions. There is an older but still relevant study of STR distribution in the human genome suggesting their non-random distribution in protein coding genes.

Li, You-Chun, et al. "Microsatellites within genes: structure, function, and evolution." *Molecular biology and evolution* 21.6 (2004): 991-1007.

We characterize fSTR distribution within introns, exons, UTRs, and coding sequences (figure 1A). We also reiterate that we have expanded characterization of fSTR and non-fSTR in the supplementary material of the manuscript.

The study relies heavily on computational predictions, and the lack of experimental validation is a notable weakness. Incorporating experimental data to support the computational findings would strengthen the overall conclusions and provide additional credibility to the results. If not provide, please suggest experimental design of verification in the discussion.

We do rely heavily on computational prediction; but, our findings are not entirely without experimental support. All of the STR array length variants were identified in whole exome sequencing data from the 1000 Genomes Project. In other words, we do not predict secondary structures for hypothetical variants (with the exception of figure 5). In addition, RNA-seq data from 1000 Genomes Project samples were used to test for association with gene expression. So, a fair amount of experimental data is used.

We do not have experimental data that verifies the secondary structures predicted by bpRNA and bpRNA-align. However, those tools were calibrated using experimental data in the bpRNA-1m database: a collection of 102,318 RNA Secondary Structures from 7 different sources [1]. Benchmarking information is available in the bpRNA-align publication [2] which we summarize here:

Dataset	bpRNA-align Accuracy	RNAforester Accuracy	BEAGLE Accuracy
Riboswitch	1	0.92	0.75
MicroRNA	1	0.95	1
Three-Segment	0.95	1	0.89
Four-Segment	1	1	0.94
Combined	0.96	0.93	0.89

To experimentally validate our predictions regarding the impact of transcribed STR variants on RNA-protein interactions and translation efficiency, several high-resolution techniques could be employed. Crosslinking and immunoprecipitation (CLIP)-based methods, such as HITS-CLIP, PAR-CLIP, or eCLIP, can be used to directly assess changes in RNA-binding protein (RBP) occupancy at variant sites. These techniques capture in vivo protein-RNA interactions at nucleotide resolution and would allow testing whether specific STR variants alter RBP binding landscapes. Additionally, ribosome profiling (Ribo-seq) can be leveraged to measure translation efficiency genome-wide by comparing ribosome-protected fragment density across transcripts harboring different variants. Combining Ribo-seq with matched RNA-seq would enable estimation of translation efficiency as a function of ribosome occupancy normalized to transcript abundance. Together, these approaches would provide complementary insights into how sequence variants modulate post-transcriptional regulation and could serve to substantiate the computational predictions presented here.

Ideally, CLIP and/or Ribo-seq would be performed on the same 1000 Genomes Project samples as our RNA-seq dataset to confidently attribute differences in RBP binding or translation efficiency to the underlying fSTR variants and associated expression changes. This would help elevate our findings from association to causation.

While we recognize the value of experimental validation, the implementation of these assays requires specialized infrastructure, technical expertise, and funding that is currently beyond the capabilities of our institution. As such, these experiments are not feasible to perform in-house at this time. We view the

present work as a foundation for hypothesis generation, and we welcome future collaborations or studies in better-equipped research settings.

[1] Danaee, Padideh, et al. "bpRNA: large-scale automated annotation and analysis of RNA secondary structure." *Nucleic acids research* 46.11 (2018): 5381-5394.

[2] Lasher, Brittany, and David A. Hendrix. "bpRNA-align: improved RNA secondary structure global alignment for comparing and clustering RNA structures." *RNA* 29.5 (2023): 584-595.

Reviewer #3:

This manuscript presents a compelling and timely investigation into the role of short tandem repeat (STR) variants in influencing RNA secondary structure and their potential downstream effects on gene expression. The authors integrate data from the 1000 Genomes Project with RNA structure predictions using ViennaRNA, and associate RNA structural alterations with expression profiles from Geuvadis RNA-seq data. The identification of 17,255 fSTRs, including 356 with significant expression associations, contributes meaningfully to our understanding of regulatory variation beyond SNPs.

We appreciate the positive feedback.

Experimental Validation: While the computational predictions are strong, the manuscript would benefit from even a brief discussion of possible experimental strategies to validate the RNA structure changes, such as SHAPE-seq or DMS-seq.

It's a good idea to suggest experimental validation; the same suggestion was made by another reviewer. We have added the following passage to our discussion:

> To validate our computational predictions regarding RNA-protein interactions and translation efficiency, several experimental techniques could be employed. Cross-linking immunoprecipitation (CLIP) methods, such as HITS-CLIP or iCLIP, allow for transcriptome-wide mapping of protein binding sites on RNA at nucleotide resolution. Applying CLIP to our system would test whether predicted RNA variants alter protein binding in vivo. Similarly, SHAPE-seq and DMS-seq could provide experimental insight into RNA secondary structure changes caused by fSTR variants.
>
> For translation efficiency, ribosome profiling (Ribo-seq) offers a powerful means to assess ribosome occupancy along transcripts. Comparing ribosome footprint density across transcript variants could determine if fSTR variants influence translation in vivo. When used in parallel with RNA-seq from the same samples, Ribo-seq also enables calculation of translational efficiency ratios, providing a direct test of our predictions. Together, these approaches offer complementary validation strategies that could substantiate the functional effects of fSTRs proposed in this study.

Functional Implications: The discussion could be expanded to address how fSTR-induced RNA structural changes might mechanistically influence RNA processing, stability, or translation beyond splicing—particularly for those fSTRs located in UTRs or coding regions.

We have updated the discussion with the following passage:

> Beyond splicing, RNA secondary structure influences post-transcriptional gene regulation, particularly when variants occur in untranslated regions (UTRs) or coding sequences. Variants in the 5′ UTR may modulate translation initiation while those in coding may affect elongation rates. Variants in the 3′ UTR may impact transcript stability or localization by disrupting motifs for RNA-binding proteins. Future work integrating ribosome profiling, RNA stability assays, and RBP mapping will help clarify and validate the broader functional consequences of fSTRs.

Statistical Considerations: It would be helpful if the authors described any multiple testing corrections applied during the association analysis between cluster assignments and gene expression. A false discovery rate threshold would bolster confidence in the reported 356 significant fSTRs.

This issue was taken into serious consideration. We consulted with a statistician who recommended Tukey's Honestly Significant Difference (HSD) test. Essentially, we end up testing all pairwise differences between means for RPKM values sorted into their cluster assignments (figure 1). Some of this was documented in the first paragraph of our methods; we now have a dedicated section for statistical considerations:

> To evaluate pairwise differences between binned datapoints, we used Tukey's Honestly Significant Difference (HSD) test, which is specifically designed for post-hoc comparisons following ANOVA. This method controls the family-wise error rate (FWER), reducing the likelihood of false positives that can arise from multiple testing. Tukey's HSD achieves this by adjusting the significance threshold across all pairwise comparisons, ensuring that the overall probability of making one or more Type I errors remains at the specified alpha level (typically 0.05). As such, it provides a conservative and statistically robust approach to identify significant group differences while accounting for the multiple comparisons inherent in our analysis.
>
> While it is true that multiple testing corrections can be applied both within and across families of tests, we chose to apply Tukey's Honestly Significant Difference (HSD) test within each binned comparison group without an additional layer of correction across bins. This decision reflects our aim to identify localized effects of specific variants or sequence contexts, rather than to make broad claims about global significance across the entire dataset. Tukey's HSD already controls the family-wise error rate for the multiple pairwise comparisons within each group, which are the relevant statistical units for our hypotheses. Furthermore, because each bin represents a biologically distinct context, we treat these as independent analytical units rather than as components of a single multiple testing framework. As such, we interpret statistical significance conservatively and contextualize findings based on consistency across bins and biological plausibility, rather than relying solely on adjusted p-values for global inference.

Visualization: The figures could be improved with clearer legends and annotations, particularly for readers who may not be familiar with RNA structural clustering or exemplars.

The editor also suggested updating figure 1 for clarity. The figure no longer highlights the exemplars which distract from the main ideas: clustering of transcribed STR allele structures (figure 1A) and mapping gene expression data into those clusters based on genotype (figure 1B).

The manuscript would benefit from language editing in several places to improve clarity and flow, particularly in the abstract and introduction.

Always a good idea to continue proofreading; we have made several changes throughout the manuscript.

Please ensure all acronyms (e.g., fSTR, bpRNA) are defined at first use in the main text.

Noted.

Some references for gene expression may be helpful. Inhibitory Effect of Multimodal Nanoassemblies against Glycative and Oxidative Stress in Cancer and Glycation Animal Models. BioMed Research International. 2021 Apr 9;2021:8892156. Revisiting global gene expression analysis. Cell. 2012 Oct 26;151(3):476-82.

These are helpful indeed. We have added the second as a citation.
