# Supplementary material for: Short tandem repeat variants are possibly associated with RNA secondary structure and gene expression
Source: PLoS One. 2025 Jun 18;20(6):e0326355. doi: 10.1371/journal.pone.0326355 (PMC12176206; doi:10.1371/journal.pone.0326355)
Supplement: Attachment — Submitted filename: Response_to_reviewers_auresp_2.pdf [file pone.0326355.s004.pdf]

Nicholas Kinney, PhD
PO BOX 121
Sweet Briar, VA 24595
Phone: 301.338.1181
Email: nkinney@sbc.edu

May 27, 2025

Karthikeyan Thiyagarajan, PhD
Academic Editor, PLOS One
RE: POINT BY POINT RESPONSE TO PEER REVIEW COMMENTS (2nd REVISION)

Dear Dr Thiyagarajan:

In the pages that follow we respond point-by-point to each of the additional comments concerning our manuscript: “SHORT TANDEM REPEAT VARIANTS ARE POSSIBLY ASSOCIATED WITH RNA SECONDARY STRUCTURE AND GENE EXPRESSION”.

We appreciate the comments which have helped to improve our manuscript. You suggested a title update to emphasize that much of the work depends on computational tools. This seems very appropriate; we have updated the title and manuscript accordingly. The remaining reviewers appear satisfied with the manuscript. If needed we would like to reiterate sources of funding as requested by the journal:

• The authors received support for publication fees from the Sweet Briar College Faculty Advancement Committee

• The funders had no role in study design, data collection and analysis, decision to publish, or preparation of the manuscript.

• The authors did not receive a salary from any funders.

We are excited to share our revised manuscript with the scientific community and look forward to hearing back. There are no related articles published or under consideration. If there are any specific questions that need a conversation, my cell is 301 338-1181.

Sincerely,

Dr. Nicholas Kinney
Assistant Professor of Biology, Sweet Briar College

Editorial comments:

Most of your revisions and responses are acceptable. The manuscript is directly based on the computational prediction of short tandem repeat-based RNA folding and its impact on gene expression, etc. So, there is no experimental proof; however, you have added relevant experiment articles to support your analysis and hypothesis. I suggest please changing the title to "SHORT TANDEM REPEAT VARIANTS ARE POSSIBLY ASSOCIATED WITH RNA SECONDARY STRUCTURE AND GENE EXPRESSION". So it is understandable about the specific RNA folding due to tandem repeat variation for the possible associations with gene expressions with a prediction basis rather than the wet lab-related experimental approaches.

Thank you for the feedback and orchestrating peer reviews on our behalf. The title change seems very reasonable; we have updated accordingly.

Wherever required, please change throughout the manuscript to emphasize your results about the possibility of the associations with gene expressions rather than completely concluding the associations with the expression that may need additional lab experiments.

We have made several updates throughout the manuscript.

One of the reviewers also suggested raw data with the coordinates of the genes tested and STRs, but your response was, "We look forward to it!" Please clarify.

Perhaps our response was a bit too brief. The raw data used for analysis consists of several data frames (.tsv files) and python scripts (.py files). Since these items are rather large, they are more appropriate as supplementary material rather than the main text. We also look forward to sharing the code on github.com if the manuscript is published.

Reviewer #1: All comments have been adequately addressed, I believe the results are meaningful. The authors tried to explain in detail.

We appreciate the positive feedback.
